# Effects of Cold-Dry (Harmattan) and Hot-Dry Seasons on Daily Rhythms of Rectal and Body Surface Temperatures in Sheep and Goats in a Natural Tropical Environment

**DOI:** 10.5334/jcr.143

**Published:** 2016-11-29

**Authors:** Ndazo S. Minka, Joseph O. Ayo

**Affiliations:** 1 College of Agriculture and Animal Science, Department of Animal Health and Husbandry, P.M.B. 2134, Division of Agricultural Colleges, Ahmadu Bello University, Mando-Kaduna, Nigeria; 2 Department of Veterinary Physiology, Faculty of Veterinary Medicine, Ahmadu Bello University, Zaria, Nigeria

**Keywords:** circadian rhythm, core and surface body temperature, goats, harmattan and hot-dry season, infra-red thermometer, sheep

## Abstract

Studies on daily rhythmicity in livestock under natural conditions are limited, and there is mounting evidence that rhythm patterns differ between chronobiological studies conducted in the laboratory and studies conducted under pronounced natural seasonality. Here, we investigated the influence of cold-dry (harmattan) and hot-dry seasons on daily rhythmicity of rectal (RT) and body surface temperatures (BST) in indigenous sheep and goats under natural light-dark cycles. The RT and BST of the animals, and the ambient temperature (AT) and relative humidity (RH) inside the pen, were measured every three hours for a period of two days, twice on separate days during the hot-dry and the harmattan seasons, respectively. The AT and RH had minimum values of 16°C and 15% recorded during the harmattan and maximum values of 32°C and 46% recorded during the hot-dry season, respectively. A trigonometric statistical model was applied to characterize the main rhythmic parameters according to the single cosinor procedure. The result showed that RT and BST exhibited different degrees of daily rhythmicity, and their oscillatory patterns differed with the seasons (larger amplitude during the harmattan season than during the hot-dry season). The goats displayed greater (p < 0.05) amplitude of BST than the sheep in all seasons. The acrophases were restricted to the light phase of the light-dark cycle. The mesor of RT in goats was not affected by the season, but mesors of BST in both species were significantly higher (p < 0.05) during the hot-dry than the harmattan season. The goats had a more robust RT rhythm (70%) as compared to the sheep (56%). Overall, the results demonstrated that seasonal changes influenced considerably the daily rhythmicity of RT and BST in sheep and goats under natural light-dark cycle. Awareness of these changes may be useful in the improvement of diagnosis, treatment and prevention of diseases, and welfare and productivity of sheep and goats under cold-dry and hot-dry conditions.

## Introduction

The rhythmicity of body temperature is an important process to be studied, not only to advance knowledge of the temporal variability of thermal homeostasis, but also as a means to facilitate the study of biological rhythmicity in general. In view of the relative ease of monitoring body temperature and the robustness of its rhythm, the rhythmicity of body temperature has been widely used as an indicator of the rhythmicity of the biological clock [1,2,3]. The measurement of body temperature is an important procedure for assessment of physiological status of the animal [4] as well as an ideal indicator of its responses and adaptability to environmental stress factors [5].

Assessment of livestock production in the tropics has focused more on the adverse effect of heat stress during the hot-dry season, which is characterized by high ambient temperature and intense solar radiation, and the effect of cold-dry (harmattan) stress on livestock has been poorly investigated [7]. The harmattan season, characterized by cold-dry and dusty wind, blowing north-east and west off the Sahara desert into the Gulf of Guinea, has been shown to be the most thermally stressful season in the Northern Guinea Savannah zone [7,8,9].

Studies on daily rhythmicity of physiological variables have been conducted mostly on humans and laboratory animals, while the subjects were maintained in a thermoneutral environment or controlled experimental photoperiod [3,10]. While such studies remain relevant in understanding the endogenous and exogenous roles in producing circadian rhythms, there is mounting evidence that rhythm patterns often differ between laboratory-controlled settings and natural environment [11]. Individuals under natural conditions are involved in complex interrelation with the microenvironment, which may produce changes in circadian activities that are not reflected in purely chronobiological studies carried out under laboratory conditions [10,11]. Only of recent, few studies were conducted under natural conditions on livestock involving the influence of ambient temperature on the daily rythmicity of core temperature and blood biochemical variables [3,12,13,14,15]. The effects of natural environmental conditions on daily rhythm in livestock kept under extensive management system and pronounced natural seasonality requires further investigation.

Measurements of body surface temperature (BST) in animals with the aid of infrared thermography (IRT) and infrared thermometry (IRTM), which are non-contact methods of temperature measurements, offer several advantages over contact methods used in veterinary medicine [16,17,18]. A comprehensive collection of infrared imaging applications in livestock is described by Luzi et al. [19].

To date, there is a paucity of information in the available literature on the effect of the harmattan and hot-dry seasons on daily rhythmicity of core body temperature and body surface temperature (BST) of sheep and goats reared in the Northern Guinea Savannah zone. Studies with the aid of IRT for measurement of BST rhythmicity and thermographic standards in indigenous small ruminant species under natural tropical conditions and different photoperiods have not been reported. Such information, if obtained, may be of value to clinicians, researchers and agriculturists in the understanding of biological rhythm, and in enhancing the health status, welfare and productivity of sheep and goats under different natural conditions.

The aim of the present study was to investigate the circadian rhythmicity of rectal temperature (RT) and BST, and their characteristics in sheep and goats under different natural photoperiods and seasons.

## Materials and Methods

### Study area and management of animals

The experiment was conducted during the harmattan (November-December) and hot-dry (March-April) seasons of 2015–2016 at the Livestock Farm of the College of Agriculture and Animal Science, Mando-Kaduna (11° 10′ N, 07° 38′ E), located in the Northern Guinea Savannah zone of Nigeria. Twenty healthy, mature indigenous goats (Red Sokoto goats) and 20 indigenous sheep (Yankasa sheep), aged 1.5–2 years served as subjects. The animals were kept under a natural light-dark cycle during the hot-dry and harmattan seasons at the small ruminant research pen of the College of Agriculture and Animal Science, Mando-Kaduna, Nigeria. They were housed in individual stands in communal pens, made of concrete floor and cement block wall. The pens measured 2.42 m × 7.39 m wide and 1.12 m high of wire-mesh from the floor, which provided for adequate ventilation. The animals were fed beans husk, maize bran and groundnut hay, and given access to clean drinking water *ad libitum*. They were routinely dewormed, treated against ticks and vaccinated against most common diseases of small ruminants in the area of study. The health of the animals was assessed a week before the start of the study by screening of their blood samples for haemo-parasites, and by clinical evaluations of their rectal temperature, and respiration and heart rates for three consecutive days.

### Measurements of rectal temperature and thermal environental conditions

The ambient temperature (AT) and relative humidity (RH) inside the pen, and the RT and BST of the sheep and goats were measured on two separate days at three-hour intervals for two days. Each measurement began at 08:30 h on the first day and ended at 08:30 h the second day. The animals were easily restrained for measurements of RT, recorded from each goat as an indicator of the body temperature by a digital clinical thermometer (Electron Thermometer, COCET, Kangfu Medical Equipment, Zhejiang Yueqinq, China), inserted at about 2 cm into the rectum via the anus. The readings were taken after an alarm sound was heard, indicating the end of the measurement. Within the same period of RT measurements, BST was also recorded at five different body locations using a digital infrared thermometer (Model AR330, China) with a resolution of 0.1°C. The locations comprised the inter-digital space, coronary band, mid-head, nose and ear-base regions. The IRT was focused at an approximate distance of not more than 1.2 meters from the site of recording [20].

The dry- and wet-bulb temperature values were obtained concurrently with the RT readings at the experimental site, using a dry- and wet-bulb thermometer (Hartmann Company, England). The animals were humanely handled and treated according to Code of Recommendations for the Welfare of Livestock [21] and as approved by the Research, Seminar and Welfare Committee of the Animal Health Department of the College (No. 44–14/16).

### Statistical analysis

Each time series was evaluated for rhythmicity by repeated-measures analysis of variance (ANOVA model-3) and by the cosinor procedure [3,22]. Cosinor analysis was used to determine the RT and BST daily rhythms of individual animals. The mean mesor (rhythm-adjusted mean), amplitude (half the range of excursion or a measure of the extent of predictable change within a cycle), acrophase (time of peak) and robustness (strength of rhythmicity computed as the fraction of the variance explained by the cosine model) values of the variables of daily rhythm were calculated for each animal and for each time series of the study period. Rhythm robustness was computed as a percentage of the maximal score, attained by the chi-square periodogram statistic for ideal data sets of comparable size and 24-h periodicity. The effects of animal species, season and interaction between seasons and time of day within subjects were compared. The difference was tested using two-way repeated-measures ANOVA. Values of p < 0.05 were considered significant.

## Results

### Ambient temperature and relative humidity

**Figure 1:**
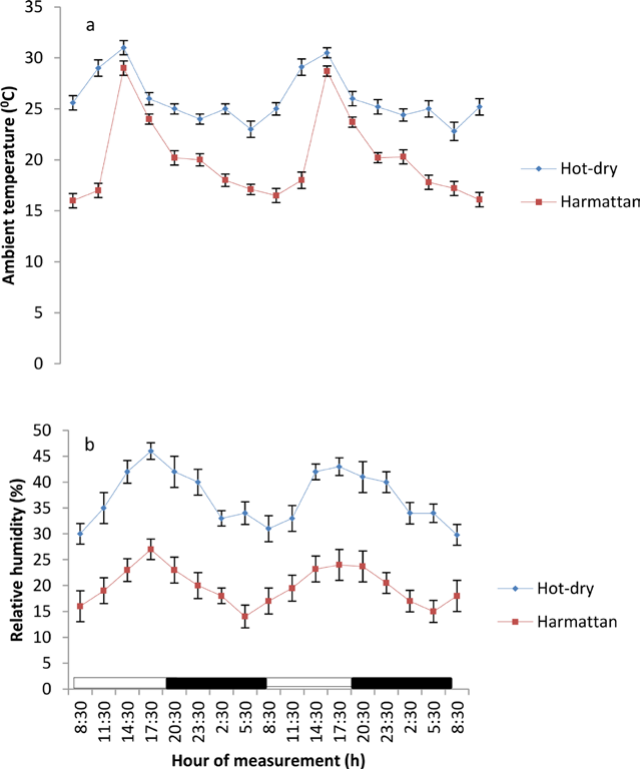
Fluctuatios in ambient temperature **(a)** and relative humidity **(b)** inside the animals pen during the hot-dry and harmattan seasons.

The AT and RH during the study periods are depicted in Figures 1a and b. The mean, maximum and minimum AT values of 28.4 ± 0.2, 32.5 and 23.0°C recorded during the hot-dry season were higher (P < 0.05) than the corresponding values of 20.1 ± 0.1, 28.2 and 16°C, obtained during the harmattan season (Figure 1a and b). A similar trend was observed in the RH values (Figure 1b). In general, the AT and RH values were lower during the scotophase than the photophase.

### Rectal temperature

The mean, maximum and minimum RT values obtained during the study period in the goats were 38.65°C ± 0.05, 39.5°C and 37.8°C, respectively; and in sheep the values were 38.14 ± 0.05°C, 39.20°C and 37.0°C, respectively.

**Figure 2:**
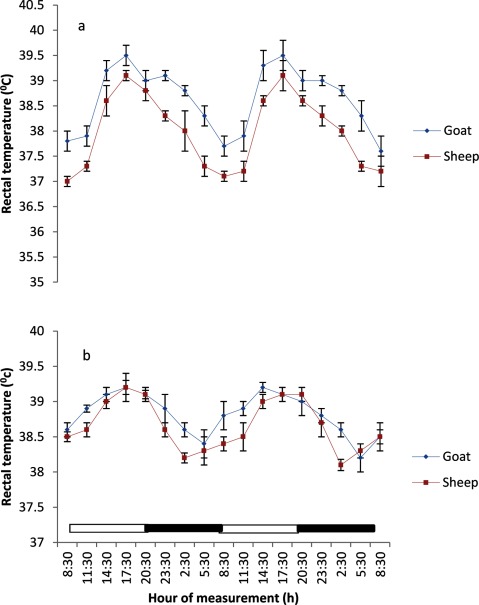
Effects of harmattan **(a)** and hot-dry **(b)** seasons on daily rhythms of rectal temperature in sheep and goats. Notes: Each data point represents the mean ± SEM of 20 animals at each period of measurements. Rectal temperature was clearly low during the scotophase and rises during the photophase, especially during the harmattan season. The horizontal bars denote the dark and light phases of the prevailing light-dark cycle. For each season measurements were made twice on separate dates at 3-h intervals for a period of 2 days.

In both seasons the RT of the sheep and goats exhibited a strong and clear daily rhythm (Figures 2a and b). Higher peak values of the daily rhythm of RT were recorded in both sheep and goats during the photophase (07:00–19:00 h) as compared to scotophase.

### Characteristics of rectal temperature

Table 1 shows the RT rhythmic parameters of mesor, amplitude, acrophase and robustness. During the harmattan in sheep, the mesor (38.2) was lower, and the amplitude was greater (1.20); while the acrophase was delayed to 20:00 h as compared with the mesor value of 38.8, amplitude of 0.85 and acrophase of 17:30 h observed in goats. During the hot-dry season, the rhythmic parameter of mesor in sheep (38.8 ± 0.5°C) was not different from the value obtained in goats (38.7 ± 0.3°C). The amplitude values were slightly different (0.55 in sheep versus 0.45 in goats). Sheep and goats attained the acrophase (highest peak) at the same time (18:00 h). The goats had a more robust RT rhythm (70%) as compared to the sheep (56%) (Table 1).

**Table 1:** Characteristics of rectal temperature and body surface temperature daily rhythms in sheep and goats under natural dark:light circle during the harmattan and hot-dry seasons. For each season measurements were made twice on separate dates at 3-h intervals for a period of 2 days.

	Harmattan Season		Hot-dry Season	

	Goat (n=20)	Sheep (n=20)	Goat (n=20)	Sheep (n=20)

**Mesor (°C):**				
Rectal temperature	38.8 ± 0.2^a^	38.2 ± 0.3^b^	38.8 ± 0.5^a^	38.7 ± 0.4^a^
Inter-digital space	22.7 ± 0.4^a^	22.0 ± 0.2^a^	27.4 ± 0.1^b^	26.5 ± 0.5^b^
Coronary band	22.5 ± 0.4^a^	21.4 ± 0.5^a^	27.6 ± 0.2^b^	26.1 ± 0.4^b^
Nose	24.1 ± 0.2^a^	23.4 ± 0.4^a^	29.0 ± 0.4^b^	28.7 ± 0.5^b^
Head	27.7 ± 0.3^a^	26.1 ± 0.2^b^	31.2 ± 0.5^c^	30.8 ± 0.2^c^
Ear	27.2 ± 0.2^a^	25.0 ± 0.5^b^	30.7 ± 0.4^c^	30.7 ± 0.4^c^
**Amplitude (°C):**				
Rectal temperature	0.85 ± 0.02^a^	1.20 ± 0.05^b^	0.55 ± 0.01^c^	0.45 ± 0.04^c^
Inter-digital space	5.60 ± 0.10^a^	6.35 ± 0.20^b^	4.51 ± 0.10^c^	3.01 ± 0.02^d^
Coronary band	4.60 ± 0.01^a^	3.50 ± 0.04^b^	4.50 ± 0.07^a^	3.50 ± 0.04^b^
Nose	7.15 ± 0.20^a^	6.65 ± 0.08^b^	5.30 ± 0.01^c^	4.10 ± 0.02^d^
Head	5.65 ± 0.08^a^	5.25 ±0.04^a^	4.90 ± 0.02^b^	4.80 ± 0.06^b^
Ear	7.00 ± 0.21^a^	6.00 ± 0.11^b^	5.50 ± 0.05^c^	5.00 ± 0.05^c^
**Acrophase (h):**				
Rectal temperature	17:30 ± 0.14^a^	20:30 ± 0.10^b^	18:00 ± 0.12^c^	18:00 ± 0.14^c^
Inter-digital space	17:30 ± 0.20^a^	17:30 ± 0.11^a^	11:30 ± 0.10^b^	11:30 ± 0.10^b^
Coronary band	17:30 ± 0.25^a^	17:30 ± 0.10^a^	11:30 ± 0.11^b^	11:30 ± 0.10^b^
Nose	17:30 ± 0.15^a^	17:30 ± 0.14^a^	11:30 ± 0.10^b^	11:30 ± 0.10^b^
Head	17:30 ± 0.15^a^	14:30 ± 0.15^b^	11:30 ± 0.10^c^	11:30 ± 0.11^c^
Ear	17:30 ± 0.14^a^	14:30 ± 0.12^b^	11:30 ± 0.11^c^	11:30 ± 0.12^c^
**Robustness (%)**				
Rectal temperature	69 ± 10.5^a^	54 ± 12.7^b^	71 ± 15.2^a^	58 ± 10.5^b^
Inter-digital space	60 ± 9.5^a^	52 ± 11.5^a^	61 ± 8.7^a^	58 ± 11.2^a^
Coronary band	59 ± 10.0^a^	55 ± 9.5^a^	60 ± 10.2^a^	60 ± 7.5^a^
Nose	54 ± 7.8^a^	44 ± 8.6^a^	50 ± 9.5^a^	48 ± 10.0^a^
Head	52 ± 8.5^a^	50 ± 9.2^a^	60 ± 10.5^a^	55 ± 11.4^a^
Ear	60 ± 12.4^a^	58 ± 10.2^a^	62 ± 8.9^a^	58 ± 7.3^a^

Notes: Mean values with different superscript letters along the same row are significantly different at *p* < 0.05.

### Body surface temperatures

**Figure 3:**
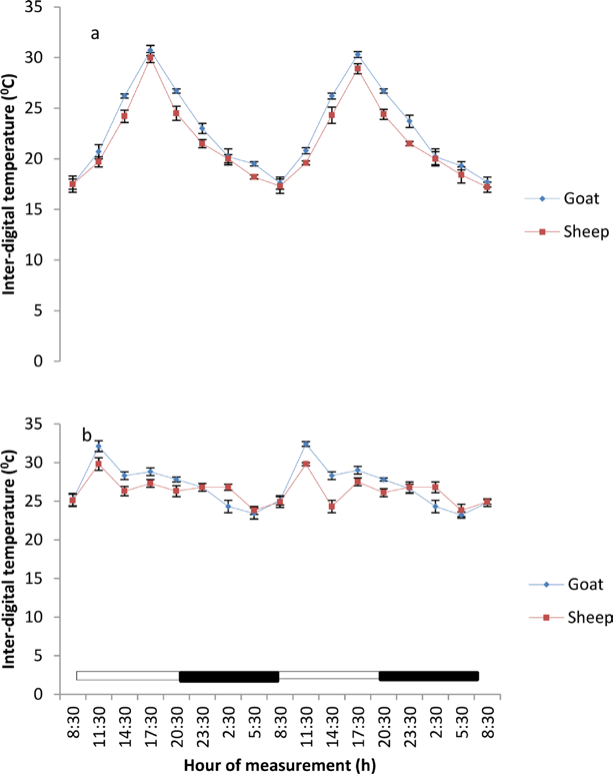
Effects of harmattan **(a)** and hot-dry **(b)** seasons on daily rhythms of inter-digital temperature in sheep and goats. Notes: Each data point represents the mean ± SEM of 20 animals at each period of measurements. Inter-digital temperature was clearly low during the scotophase and rises during the photophase, especially during the harmattan season. The horizontal bars denote the dark and light phases of the prevailing light-dark cycle. For each season measurements were made twice on separate dates at 3-h intervals for a period of 2 days.

**Figure 4:**
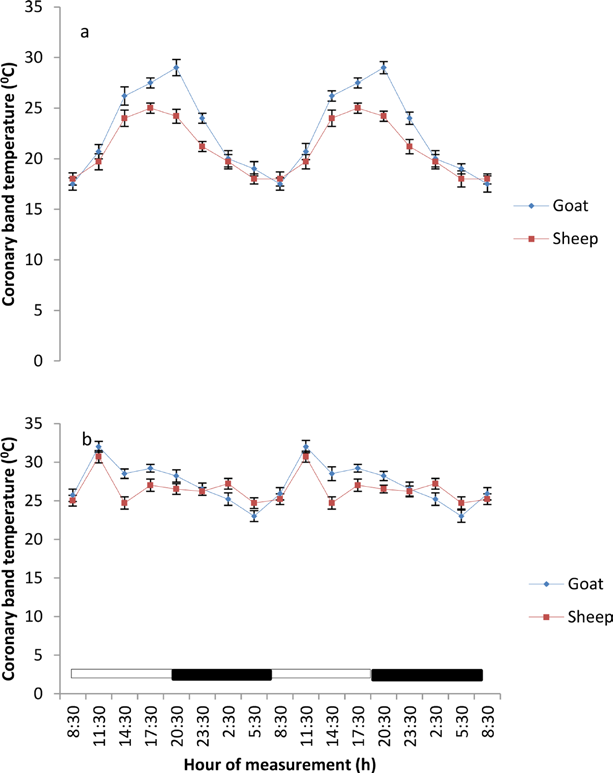
Effects of harmattan **(a)** and hot-dry **(b)** seasons on daily rhythms of coronary band temperature in sheep and goats. Notes: Each data point represents the mean ± SEM of 20 animals at each period of measurements. Coronary band temperature was clearly low during the scotophase and rises during the photophase, especially during the harmattan season. The horizontal bars denote the dark and light phases of the prevailing light-dark cycle. For each season measurements were made twice on separate dates at 3-h intervals for a period of 2 days.

**Figure 5:**
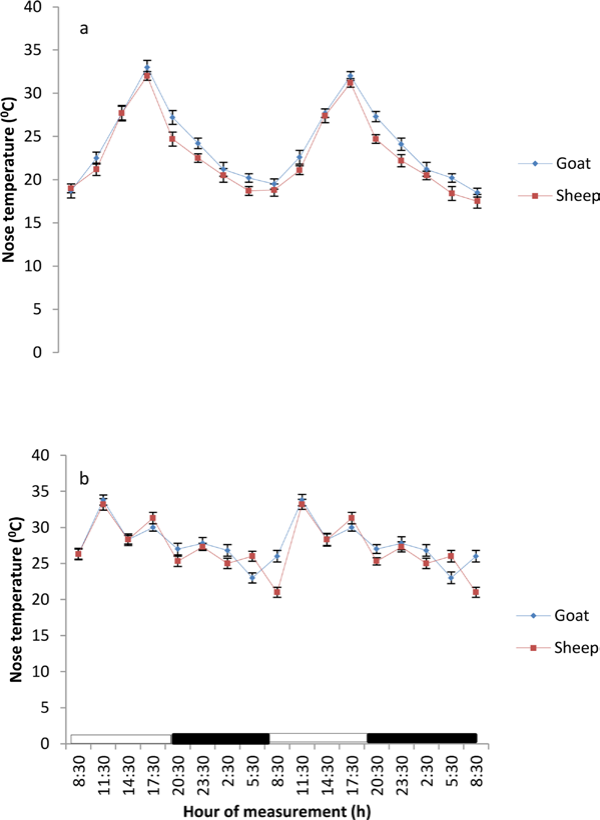
Effects of harmattan **(a)** and hot-dry **(b)** seasons on daily rhythms of nose temperature in sheep and goats. Notes: Each data point represents the mean ± SEM of 20 animals at each period of measurements. Nose temperature was clearly low during the scotophase and rises during the photophase, especially during the harmattan season. The horizontal bars denote the dark and light phases of the prevailing light-dark cycle. For each season measurements were made twice on separate dates at 3-h intervals for a period of 2 days.

**Figure 6:**
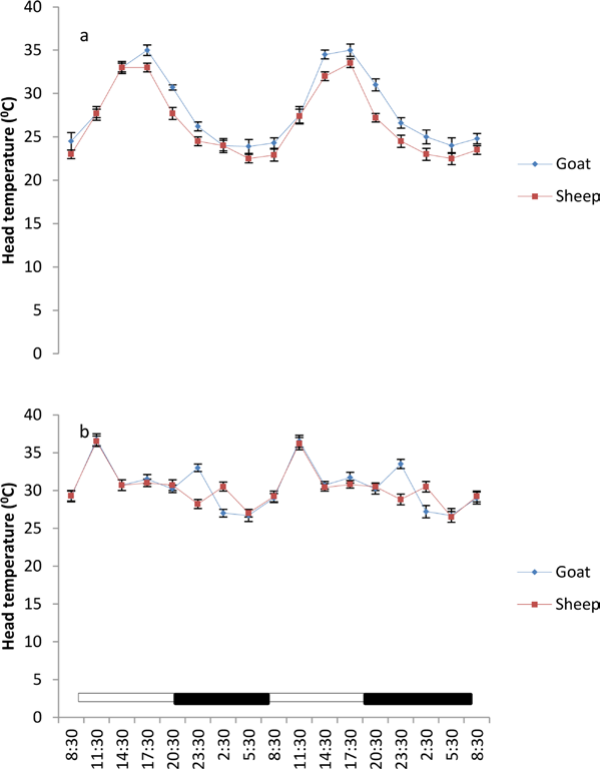
Effects of harmattan **(a)** and hot-dry **(b)** seasons on daily rhythms of head temperature in sheep and goats. Notes: Each data point represents the mean ± SEM of 20 animals at each period of measurements. Head temperature was clearly low during the scotophase and rises during the photophase, especially during the harmattan season. The horizontal bars denote the dark and light phases of the prevailing light-dark cycle. For each season measurements were made twice on separate dates at 3-h intervals for a period of 2 days.

**Figure 7:**
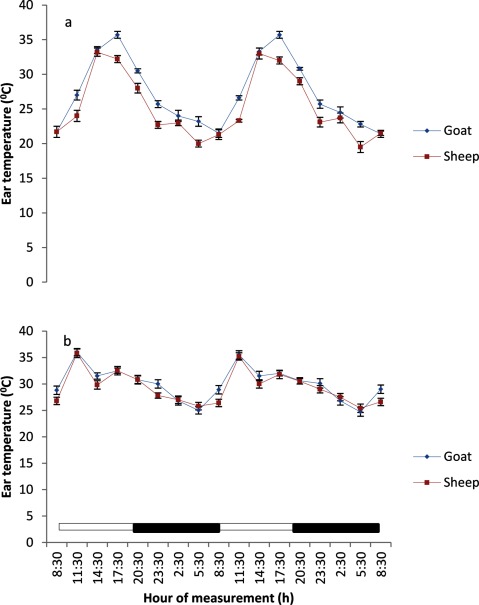
Effects of harmattan **(a)** and hot-dry **(b)** seasons on daily rhythms of ear temperature in sheep and goats. Notes: Each data point represents the mean ± SEM of 20 animals at each period of measurements. Ear temperature was clearly low during the scotophase and rises during the photophase, especially during the harmattan season. The horizontal bars denote the dark and light phases of the prevailing light-dark cycle. For each season measurements were made twice on separate dates at 3-h intervals for a period of 2 days.

The BST at various regions of the body in both sheep and goats exhibited a daily, but weak rhythmicity (Figures 3, 4, 5, 6, 7). The feet (inter-digital and coronary band) temperatures (Figure 3 and 4) were lower (p < 0.05) than those recorded in other regions, while the highest values were obtained in the head, ear and nose regions (Figure 5 and 7). The BST value differed based on the season and animal’s species. It was generally lower during the harmattan season, especially in the night, than in the hot-dry season; and in the afternoon hours of the day. Overall, the BST of goats was significantly (p < 0.05) higher than that of the sheep in all the seasons.

### Characteristics of body surface temperature

The mesors of BST recorded in both sheep and goats were significantly higher (p < 0.05) during the hot-dry than harmattan season (Table 1). In general, the extent of predictable changes (amplitudes) of the daily rhythm of BST in the sheep and goats was greater (p < 0.001) during the harmattan season as compared to the hot-dry season. The goats had greater (p < 0.05) amplitude in all the seasons. The acrophases of daily rhythm of BST were all restricted to the photophase (Table 1) with peak at 11:30 h in both sheep and goats during the hot-dry season; while during the harmattan season, the acrophases of BST coincided in time near those of the RT (17:30 h). Exceptions were the acrophases of the head and ear in sheep, which were recorded at 14:30 h. The robustness of the RT and BST rhythms of the goats and sheep is shown in Table 1. The robustness of RT was significantly (p < 0.05) stronger than the BST in all seasons. The goats had a more robust RT rhythm (70%) as compared to the sheep (56%). The seasons had no significant effect on robustness.

## Discussion

The mean AT and RH recorded in the present study in both seasons were within the thermoneutral zone of 22 – 35°C and 28 – 60%, respectively, established for sheep and goats [5,23,24]. However, the minimum AT (16°C) recorded during the scotophase of the harmattan season was outside the thermoneutral zone established for goats and sheep in the tropical regions.

The mean RT values recorded were within the normal reference intervals values of 38 – 40°C for sheep and goats in the tropics [5,8]. However, the RT values recorded in both species during the early morning hours of the harmattan season were considerably lower, which suggests that the season induced hypothermia in the sheep and goats. The result supports several reports that different seasons of the year exert different effects on body core temperature [4,25]. The RT and BST in both ruminant species exhibited individual variation typical of mammals [3]. Overall, at each hour of measurements, the RT and BST of sheep were lower than those of goats in both seasons, apparently due to species variation.

The application of the periodic model and the fitting of cosinor procedure showed that the RT exhibited a strong daily rhythmicity, while the BST exhibited a weak daily rhythmicity in both sheep and goats in all the seasons. The result agrees with the previous finding of diurnal rhythm in the RT of Comisana sheep [3] and Red Sokoto goats recorded only during the day time by Ayo *et al.* [5]. The oscillatory pattern in RT and BST differed with seasons, with stronger daily oscillation being observed during the harmattan season than during the hot-dry season.

The mesors of RT recorded in sheep and goats were consistently higher than those of BST recorded at various sites of the body, which agreed with the findings of Piccione et al. [3], who showed that surface body temperature was lower than core temperature in sheep, and this result is true of all endothermic animals [4,26]. The lower mesor of RT recorded in sheep when the AT was 16 – 28°C during the harmattan season was in contrast to that recorded by Piccione et al. [3], who found no effect of cold AT (9°C) or thermoneutral AT on mesor in sheep. The present result demonstrated the adverse effect of the harmattan season on the thermoregulation of sheep, and confirmed the earlier report that the harmattan season is the most thermally stressful of all the seasons prevailing in the Northern Guinea Savannah zone [5,7]. Although the AT (16 – 28.2°C) during the harmattan season was not as low as 9°C, its hypothermic effect on daily rhythmicity in RT of sheep was more severe than that recorded in Comisana sheep, exposed to cold AT of 9°C. The present finding suggests that, aside from breed differences, additional factors, including cold-dry air, heavy dust-laden particles and wide fluctuations in AT, which are characteristics of the harmattan season, may be responsible for the hypothermic effect.

The mesors of daily rhythm of BST in both sheep and goats demonstrated that they were modulated by AT; with higher values occurring during the hot-dry season than harmattan season, and the goats had higher mesors in all the body sites as compared to those of the sheep which had cooler BST. In both sheep and goats, the feet (inter-digital and coronary) temperatures were cooler than the RT and temperatures in other parts of the body measured, while the highest temperatures recorded in the head, ear and nose showed that these parts of the body were the hottest. The feet temperature in the present study varied compared with the finding of Piccione et al. [3], who showed that feet temperature was 9°C cooler than the rectum; however, the head temperature was similar to the value of 9°C obtained by Piccione et al. [3]. The lower feet temperature, especially during the harmattan (cold-dry) season, as compared to other sites confirmed that the feet play a more crucial role in thermoregulation than other parts of the body, probably because vasoconstriction in the feet is a good mechanism of heat conservation [3,27].

In general, the lower mesor in BST in sheep than in goats may be due to species difference, integumentary variations and body fat. In addition, the white hair-skin colour characteristic of the Yankasa sheep has been shown to influence evaporative cooling and repel heat; unlike the dark-brown colour of the goats’ skin, which may enhance solar absorption and, thus, increase heat load on the skin surface [28,29].

The greater amplitude of RT and BST during the harmattan season may be a result of the adverse effect of cold AT and greater variation of AT on the animals. Greater amplitude of core temperature has been descrbed in mammals reared under cold conditions in temperate regions [3,6,30,31].

In homeothermy, the limit of variability for amplitude of daily rhythmicity of core temperature is considered to be ± 2°C [32], which is wider than the amplitude of daily rhythmicity of RT values of 0.6 – 1.2°C recorded in sheep and goats in the present study. However, the amplitude obtained in the current study is similar to those of 0.3 – 1.2°C and 0.4 – 1.9°C, reported in sheep [33] and goats [5], respectively, but greater than the amplitudes of 0.2 – 0.4°C recorded in sheep [3,34] and 0.26 – 0.33°C in humans [35]. In both studies in sheep and goats mentioned above and in the current study, the highest amplitude of daily rhythmicity of RT values was recorded when AT oscillated about 20°C, which is also similar to the finding of Lovegrave and Heldmaier [36]. The amplitude of daily rhythmicity of BST obtained at various sites in the present study was between 3 – 7°C, which was greater than the amplitude values of 0.5 – 1.3°C in sheep [37]. The differences in the amplitudes may be due to variations in animal species and AT.

The RT during both harmattan and hot-dry seasons in the sheep and goats peaked earlier (17:30–20:00 h) than the acrophase value of 21:38 h reported in sheep [34]. During the hot-dry season, the acrophase of BST at various sites in both sheep and goats were recorded in the morning (11:30 h), but during the harmattan season the acrophase of BST was delayed till late afternoon, at about 17:30 h (8 hours after sunrise).

The relationship between RT and BST of the sheep and goats recorded during the hot-dry season showed that the BST, especially the feet temperature, reached acrophase about six hours before RT, whereas during the cold-dry (harmattan) BST peaked at the same (17:30 h) time as RT (17:30 h). The present result is in contrast with the finding of Picconne et al. [3], who obtained a peak in foot temperature six and 13 hours before RT in sheep kept under cold condition (9°C) and themoneutral zone (25°C), respectively. Our result also disagrees with the finding in humans that foot and core temperatures attained their peaks at 12 h apart [37,38]. The finding that BST, especially the feet temperature, peaked several hours before RT suggests that the mechanism of thermoregulation in the feet may be involved in production of the oscillation in RT as earlier suggested [35,38]. In the present study, however, ambient temperature fluctuated daily, thus introducing a confounding factor not present in laboratory studies.

The maximal robustness of the RT and BST rhythms recorded in the sheep and goats in the present study did not differ between seasons, although the oscillation of BST was less robust than in RT and the goats had a relatively stronger robustness than the sheep. Similar robustness values of 44–70% were reported for goats, sheep, horses [34,39,40] and dogs [41] in temperate regions. However, the robustness obtained in the present result was less than the maximal robustness of 90% and 81% recorded in cattle [42] and dromedary camels [43]. The robustness of BST obtained in the present study was similar to those reported in the limbs of sheep [34], but less than the robustness of 32% reported in camel’s skin [43].

The present result on BST indicates that IRT may be a useful non-invasive and accurate tool to detect the variations in BST in small ruminants, and the differences in BST distribution may be used for diagnosis of diseases; especially diseases accompanied with inflammatory conditions, leg disorders and detection of skin injuries, and topical applications of therapeutic agents. Overall, the present results showed that exogenous environmental cues, including seasonal variations and light-dark cycles, provide important information for adjusting body temperature in sheep and goats towards the maintenance of a stable thermoregulation.

In conclusion, the results of the present study demonstrated the effects of seasonal changes on the daily rhythmicity in RT and BST and their special features in indigenous sheep and goats under natural light-dark cycle. The data may serve as additional normal reference values for sheep and goats under different natural climatic conditions, and may be useful for the diagnosis, treatment and prevention of diseases. They may also be beneficial in the evaluation of the welfare and productivity of small ruminants under different environmental conditions.
